# β‐Catenin/c‐Myc Axis Modulates Autophagy Response to Different Ammonia Concentrations

**DOI:** 10.1002/adbi.202400408

**Published:** 2025-01-11

**Authors:** S. Sergio, B. Spedicato, G. Corallo, A. Inguscio, M. Greco, D. Musarò, D. Vergara, A. F. Muro, G. De Sabbata, L. R. Soria, N. Brunetti Pierri, M. Maffia

**Affiliations:** ^1^ Laboratory of Clinical Proteomic “V Fazzi” Hospital Lecce 73100 Italy; ^2^ Laboratory of General and Human Physiology Department of Experimental Medicine University of Salento Lecce 73100 Italy; ^3^ Laboratory of General and Human Physiology Department of Biological and Environmental Sciences and Technologies University of Salento Lecce 73100 Italy; ^4^ International Centre for Genetic Engineering and Biotechnology Trieste 34149 Italy; ^5^ Telethon Institute of Genetics and Medicine Pozzuoli 80078 Italy; ^6^ Department of Translational Medicine Federico II University Naples 80138 Italy

**Keywords:** autophagy, c‐Myc, hyperammonemia, liver

## Abstract

Ammonia a by‐product of nitrogen containing molecules is detoxified by liver into non‐toxic urea and glutamine. Impaired ammonia detoxification leads to hyperammonemia. Ammonia has a dual role on autophagy, it acts as inducer at low concentrations and as inhibitor at high concentrations. However, little is known about the mechanisms responsible for this switch. Wnt/β‐catenin signalling is emerging for its role in the regulation of ammonia metabolizing enzymes and autophagosome synthesis through c‐Myc. Here, using Huh7 cell line, we show a modulation in c‐Myc expression under different ammonia concentrations. An increase in c‐Myc expression and in its transcriptional regulator β‐catenin was detected at low concentrations of ammonia, when autophagy is active, whereas these modifications were lost under high ammonia concentrations. These observations were also recapitulated in the livers of spf‐ash mice, a model of constitutive hyperammonaemia due to deficiency in ornithine transcarbamylase enzyme. Moreover, c‐Myc‐mediated activation of autophagy plays a cytoprotective role in cells under ammonia stress conditions as confirmed through the pharmacological inhibition of c‐Myc in Huh7 cells treated with low ammonia concentrations. In conclusion, the unravelled role of c‐Myc in modulating ammonia induced autophagy opens new landscapes for the development of novel strategies for the treatment of hyperammonemia.

## Graphical abstract

Ammonia, detoxified by the liver into urea and glutamine, impacts autophagy differently at varying levels. Low ammonia activates autophagy via c‐Myc and β‐catenin, while high levels suppress it. Using Huh7 cells and *Spf‐ash* mice, c‐Myc's role in cytoprotective autophagy is revealed, offering insights into hyperammonemia and potential therapeutic strategies.

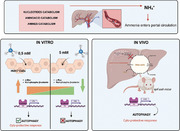

## 1 Introduction

Autophagy is a highly conserved catabolic process through which cytoplasmic components and damaged organelles are delivered to lysosomes for degradation.^[^ ^1^ ^]^ Under various stress conditions, autophagy is crucial for conferring cytoprotective effects and promoting cell survival.

Several types of stress and damage stimuli, such as the deprivation of nutrients, the damage of organelles, the unfolding and aggregation of proteins, and tissue injury have been shown to induce autophagy.^[^ ^2^ ^]^ Recent studies revealed that in several diseases, autophagy is also activated by ammonia.^[^ ^3^, ^4^, ^5^, ^6^, ^7^, ^8^ ^]^ Ammonia is a diffusible compound produced from the catabolism of nitrogen‐containing molecules and is efficiently converted into nontoxic urea and glutamine by the healthy liver.^[^ ^9^, ^10^ ^]^ In acquired liver diseases or inherited deficiencies of urea cycle enzymes, ammonia is poorly removed from the circulation resulting in a condition named hyperammonemia that is characterized by plasma ammonia levels ranging from 0.5 to 5 × 10^−3^ m, causing neuronal dysfunction.^[^ ^11^, ^12^, ^13^, ^14^ ^]^

The contrasting effects of 0.5 and 5 × 10^−3^ m NH4Cl on autophagy are particularly interesting. The lower concentration activates autophagy, suggesting that it may be a compensatory response to manage mild hyperammonemia. In contrast, the higher concentration inhibits autophagy, indicating that severe hyperammonemia can overwhelm the cellular systems responsible for maintaining balance and homeostasis. Moreover, in different papers the lowest concentration (0.5 × 10^−3^ m) is reported to activate autophagy whereas the highest (5 × 10^−3^ m) to inhibit it.^[^ ^15^, ^16^ ^]^

In addition to alteration in the autophagy process, ammonia induces multiple alterations in other cell types promoting pH changes, electrolyte imbalance and metabolic dysfunctions.^[^ ^17^ ^]^

At present, little is known about the molecular mechanisms responsible for this functional switch.

Wnt/β‐catenin signaling is emerging for its roles in the regulation of many aspects of liver biology, like the maintenance of the zonation of ammonia metabolizing enzymes in the adult liver.^[^ ^18^, ^19^ ^]^

β‐catenin is the key downstream effector of the canonical Wnt signaling pathway and it is an essential transcriptional co‐regulator of T cell‐specific transcription factor/lymphoid enhancer‐binding factor 1 (TCF/LEF) transcription factors to control target gene expression.^[^ ^20^ ^]^ Recent evidence also reported crosstalk among Wnt pathway and autophagy at different stages.^[^ ^21^ ^]^ β‐catenin is suggested to contain an LC3 interacting region (W/YXXI/L motif) which represents a direct target of selective autophagy degradation.^[^ ^22^ ^]^ Moreover, β‐catenin is a well‐recognized upstream activator of c‐Myc, a transcription factor that is involved in autophagosome formation in the early stages of autophagy. Hence, c‐Myc inhibition led to a defective autophagosome formation and a reduction of the clearance of essential autophagy substrates.^[^ ^23^ ^]^ However, whether ammonia perturbs autophagy flux through the β‐catenin/c‐Myc axis has not been evaluated. For this reason, we hypothesized the involvement of this pathway in the modulation of liver autophagy by ammonia. Here, we demonstrated in vitro, in the hepatocarcinoma Huh7 cell model, that low concentration of ammonia activated the autophagy process and increased the expression of c‐Myc and of its transcriptional regulator *β*‐catenin, while on the contrary, the opposite effect is observed at high concentrations of ammonia when the autophagy process is inhibited. This lets us hypothesize that c‐Myc is a molecular target by which ammonia orchestrates the autophagy process. Indeed, targeting of c‐Myc with a pharmacological inhibitor 10058‐F4 in Huh7 cells treated with low ammonia concentrations led to autophagy inhibition, similar to that observed under high ammonia concentrations. Consistent with the data in Huh7 cells treated with low concentrations of NH_4_Cl, livers of *spf‐ash* mice, a model of constitutive hyperammonemia, displayed increased expression of c‐Myc and active dephosphorylated form of *β*‐catenin compared to WT control mice, suggesting how also in vivo this axis could be involved in the modulation of autophagy by ammonia.

Overall, these results demonstrate the functional role of c‐Myc in regulating the effect of ammonia on autophagy.

## 2 Results

### 2.1 Autophagy Is Modulated by Ammonia in Huh‐7 Cell Line

**Figure 1:**
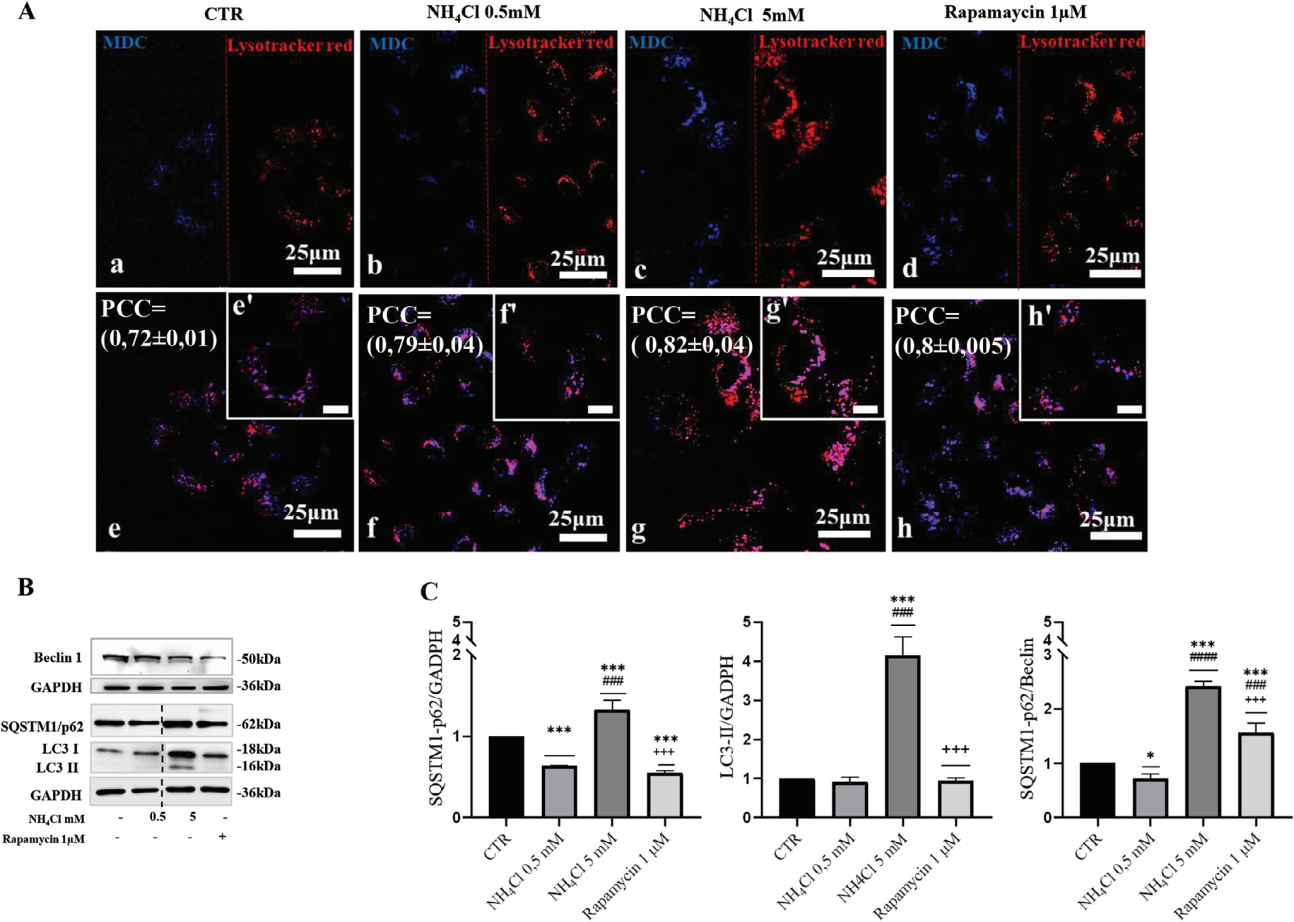
A) (a–d) Huh7 cells were cultured in presence or absence of the indicated drugs (NH_4_Cl 0.5 × 10^−3^ m; NH_4_Cl 5 × 10^−3^ m; Rapamycin 1 × 10^−6^ m) for 48 h and then stained with monodansylcadaverine to mark autophagosomes (in blue) and lysotracker red for lysosomes (in red). Scale bar is 25 µm. (e–h) Merge images from both channels are shown at the bottom where autolysosomes are shown in pink. Scale bar is 25 µm. (e′–h′) Zoom images, scale bar is 10 µm. Image correlation analysis was performed through ImageJ Jacop. PCC = Pearson's correlation coefficient. −1 < PCC < 0 = negative correlation; PCC = 0 no correlation; 0 < PCC ≤ 1 = positive correlation. The mean correlation coefficient value ± s.d. of *n* = 3 images is shown on the merge images. B) Expression levels of autophagy markers SQSTM1/p62, Beclin1, and LC3 in Huh7 cells, after 48 h of treatment with NH_4_Cl 0.5/5 × 10^−3^ m and Rapamycin 1 × 10^−6^ m. GADPH is used as loading control. Samples for the detection of SQSTM1/p62 and LC3II were run on the same gel but were not contiguous (Figure S1, Supporting Information). C) Densitometric quantification for SQSTM1/p62, LC3II, and SQSTMI:BECL1 (*n* = 3). The results were presented as means ± standard deviation; values were compared to CTR by one‐way ANOVA following Tukey test. *p < 0.0332, **p < 0.0021, ***p < 0.0002 in comparison to CTR; ^#^ *p* < 0.0332, ^##^ *p* < 0.0021, ^###^ *p* < 0.0002 in comparison to NH_4_Cl 0.5 × 10^−3^ m; ^+^ *p* < 0.0332, ^++^ *p* < 0.0021, ^+++^ *p* < 0.0002 in comparison to NH_4_Cl 5 × 10^−3^ m.

We investigated autophagy after incubation with ammonia in Huh7, a hepatic cell line derived from hepatocellular carcinoma. Toward this end, we performed staining of the cells with monodansylcadaverine to highlight autophagic vacuoles and with lysotracker red to label lysosomes. Huh7 cells were either treated with 0.5 × 10^−3^ m NH_4_Cl or with 5 × 10^−3^ m NH_4_Cl for 48 h and the two concentrations of NH_4_Cl were selected not only because they are comparable with those reported in most clinical hyperammonemia patients but also because the lowest concentration is reported to activate autophagy whereas the highest to inhibit it.^[^ ^3^, ^24^, ^25^, ^26^, ^27^, ^28^ ^]^ Cells treated with NH_4_Cl 5 × 10^−3^ m displayed an increase in autolysosomes (**Figure** **1**A panel c vs a), as shown by colocalization of monodansylcadaverine and lysotracker red (Figure 1A panel g; PCC = 0.82 ± 0.04) compared to untreated cells (Figure 1A panel e; PCC = 0.72 ± 0.01). In contrast, cells treated with 0.5 × 10^−3^ m NH_4_Cl showed increased red signal corresponding to lysosomes, blue signal corresponding to amphisomes, and pink signal corresponding to autolysosomes (Figure 1A panel f; PCC = 0.79 ± 0.04), consistent with the staining in cells treated with Rapamycin (Figure 1A panel h; PCC = 0.8 ± 0005), a well‐established autophagy inducer. By Western blot analysis, Huh7 cells treated with 5 × 10^−3^ m NH_4_Cl showed an accumulation of the autophagy cargo receptor SQSTM1/p62 and LC3‐II (autophagosome marker) compared to cells treated with 0.5 × 10^−3^ m NH_4_Cl (Figure 1B,C) that instead presented a reduction of SQSTM1/p62 and where LC3‐II band was not visible, thus suggesting high autophagic flux, as observed in Huh7 cells treated with rapamycin (a well‐known autophagy inducer) (Figure 1B,C). As alternative, also the SQSTM1:BECN1 protein level ratio can be used as a readout of autophagy.^[^ ^29^ ^]^ Since both decreased SQSTM1 levels and increased BECN1 levels correlate with enhanced autophagy, a decreased SQSTM1:BECN1 protein level ratio may be interpreted as augmented autophagy.^[^ ^30^ ^]^ Indeed, here we found a decrease in SQSTM1:BECN1 protein ratio^[^ ^29^ ^]^ following treatment with 0.5 × 10^−3^ m NH_4_Cl. On the contrary, the increase in SQSTM1:BECN1 protein level ratio in Huh7 treated with 5 × 10^−3^ m NH_4_Cl confirmed inhibition of autophagy (Figure 1B,C). Huh 7 cells treated with rapamycin 1 × 10^−6^ m did not show a reduction in the SQSTM1:BECN1 protein level ratio, due to a Beclin1 independent regulation from mTOR.^[^ ^31^ ^]^

**Figure 2:**
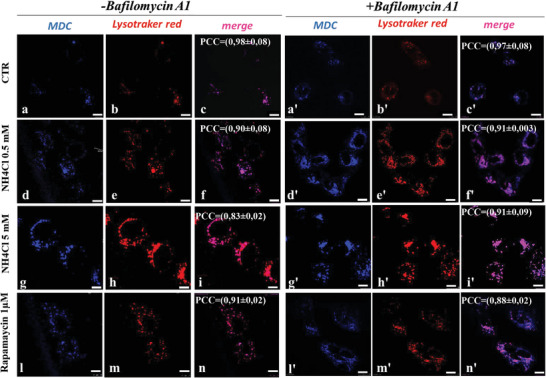
Huh7 cells were treated for 48 h with the indicated concentration of ammonia and rapamycin. Bafilomycin A1 (100 × 10^−9^ m) was added 4 h before to perform the staining with mondansylcadaverine for autophagosomes (in blue) and lysotraker red for lysosomes (in red). Scale bar is 10 µm. In panel b′, e′, h′, and m′ color scale has a maximum value of 154 A.U. against the 255 A.U. used in the other panels, to better display Lysotraker red fluorescence signal that could be affected by Bafilomycin A1 treatment. See Figure S2 (Supporting Information) for original images. Image correlation analysis was performed through ImageJ Jacop. PCC = Pearson's correlation coefficient. −1 < PCC < 0 = negative correlation; PCC = 0 no correlation; 0 < PCC ≤ 1 = positive correlation. The mean correlation coefficient value ± s.d. of *n* = 3 images is shown on the merge images.

To confirm the opposite effect of low and high concentrations of NH_4_Cl on autophagic flux, monodansylcadaverine staining was performed with or without Bafilomycin A1, a V‐ATPase inhibitor that blocks the autophagic flux by inhibiting autolysosome acidification (**Figure** **2**). Huh7 cells treated with 5 × 10^−3^ m NH_4_Cl showed increased number of autophagosomes without bafilomycin A1, with respect to CTR cells (Figure 2 panel g), that did not further increase following incubation with bafilomycin A1 (Figure 2 panel g′), suggesting a block in autophagosomes degradation. In contrast, cells treated both with 0.5 × 10^−3^ m NH_4_Cl or Rapamycin showed an increase in the number of autophagosomes that was further increased after treatment with Bafilomycin A1 (Figure 2 panel d vs d′, panel l vs l′). Altogether, these data demonstrate that lower concentration of ammonia activates autophagy whereas higher concentrations result in a block of autophagy in Huh7 cells.

### 2.2 Ammonia Modulates Autophagy through c‐Myc

**Figure 3:**
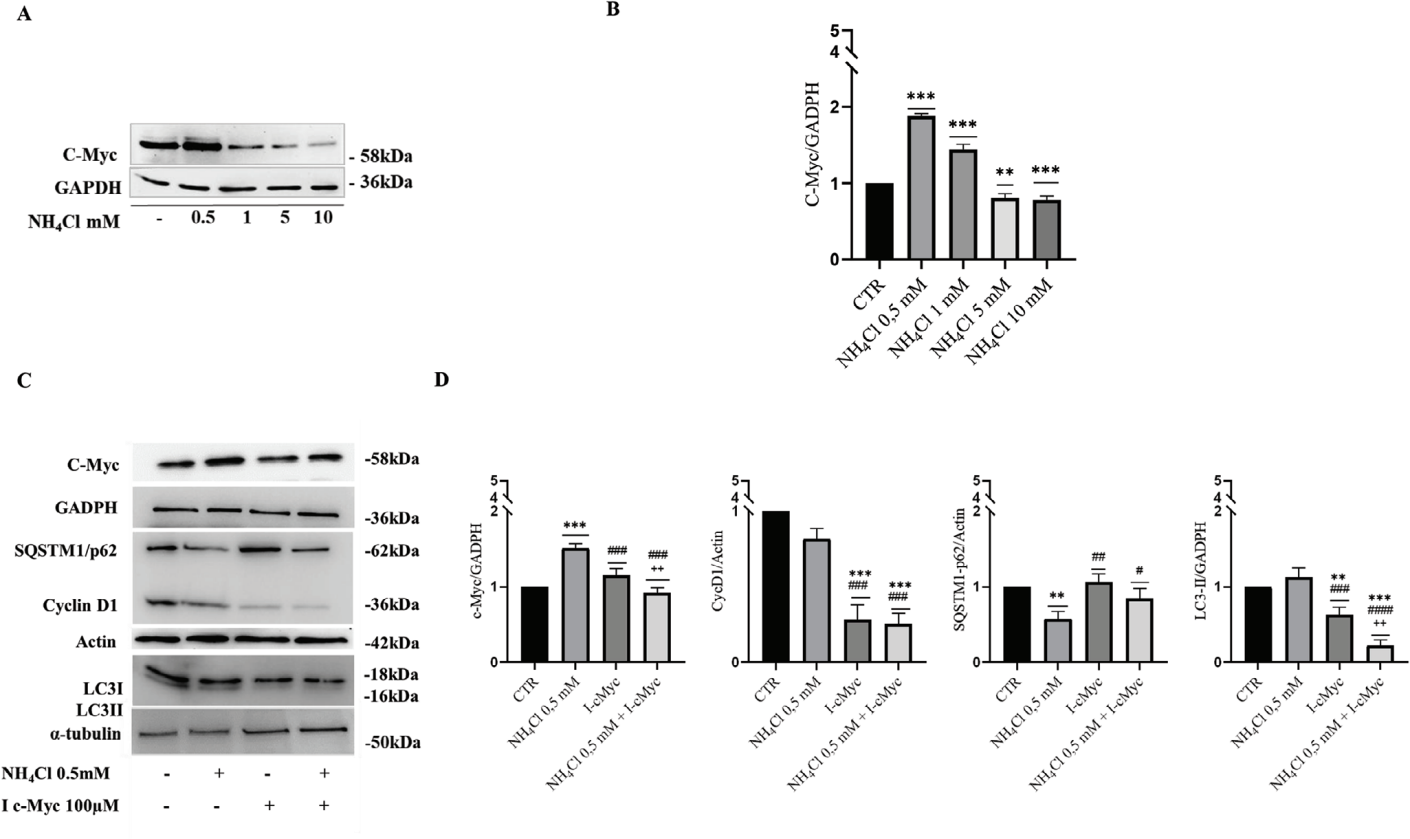
A,B) Western blot analyses and densitometric quantification of c‐Myc in Huh7 cells treated with increased concentration of NH_4_Cl for 24 h. GADPH is used as loading control (*n* = 3); C,D) Western blot analyses and densitometric quantification of c‐Myc, SQSTM1/P62, CyclinD1, LC3I/II in Huh7 cells treated with NH_4_Cl 0.5 × 10^−3^ m and/or c‐Myc inhibitor 100 × 10^−6^ m (10058‐F4) for 48 h. GADPH, actin, and α‐tubulin are used as loading control (*n* = 3). The results were presented as means ± standard deviation; values were compared to CTR by one‐way ANOVA following Tukey test, *p < 0.0332, **p < 0.0021, ***p < 0.0002 in comparison to CTR; ^#^ *p* < 0.0332, ^##^ *p* < 0.0021, ^###^ *p* < 0.0002 in comparison to NH_4_Cl 0.5 × 10^−3^ m; ^+^ *p* < 0.0332, ^++^ *p* < 0.0021, ^+++^ *p* < 0.0002 in comparison to NH_4_Cl 5 × 10^−3^ m.

Since c‐Myc, a target of the Wnt/β‐catenin pathway, is reported to regulate autophagosomes formation, we hypothesized that it could might be involved in the modulation of liver autophagy by ammonia. Having established that low ammonia concentration (NH_4_Cl 0.5 × 10^−3^ m) activates autophagy, while high ammonia concentration (NH_4_Cl 5 × 10^−3^ m) inhibits it, to demonstrate the involvement of c‐Myc in this functional switch, we first analyzed if there was a modulation in c‐Myc expression under different ammonia concentrations. Thus, Huh‐7 cells were treated with increasing concentrations of NH_4_Cl up to 10 × 10^−3^ m for 24 h that resulted in c‐Myc upregulation at 0.5 × 10^−3^ m, but in a concentration‐dependent reduction of c‐Myc at concentrations of 1 × 10^−3^ m and higher (**Figure** **3**A,B). To confirm that the increase in c‐Myc expression in NH_4_Cl 0.5 × 10^−3^ m Huh7 treated cells caused autophagy activation and instead its downregulation after NH_4_Cl 5 × 10^−3^ m treatment caused autophagy inhibition, we studied how the autophagy markers changed in Huh7 cells treated with NH_4_Cl 0.5 × 10^−3^ m, after c‐Myc inhibition. To this end, we tested a small molecule inhibitor of Myc, 10058‐F4 (referred to as c‐Myc I, hereafter) that inhibit Myc by disrupting its dimerization with its partner Max, necessary for the transactivation of target genes.^[^ ^32^ ^]^ Indeed, after the treatment with c‐Myc inhibitor we verified that its transcriptional activity was inhibited analyzing the expression of one of its target genes, CyclinD1 that resulted reduced (Figure 3C,D). Decreased LC3II and increased SQSTM1/p62 were detected in Huh7 cells treated with NH_4_Cl 0.5 × 10^−3^ m and/ or c‐Myc I, thus suggesting the inhibition of the autophagy process, with respect to Huh7 cell treated with only NH_4_Cl 0.5 × 10^−3^ m where modulation of autophagy markers LC3II and SQSTM1/p62 confirmed autophagy activation, as previously demonstrated (Figure 3C,D).

**Figure 4:**
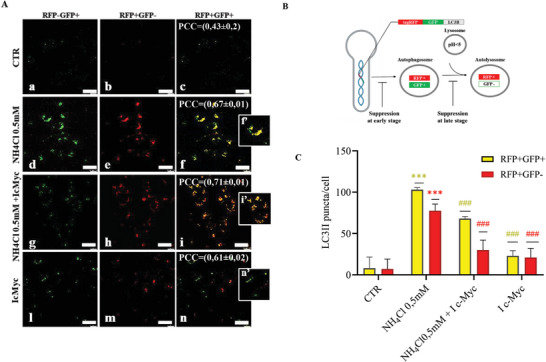
A) (a–n) Huh7 cells were transduced with 40 particles per cell of the Premo Autophagy Tandem Sensor and incubated overnight ≥16 h. Cells were then incubated with NH_4_Cl 0.5 × 10^−3^ m and/or c‐Myc I for 48 h. Cells were imaged using standard FITC/TRITC filters sets. Scale bar: 25 µm. f′, i′, n′ are zoom images. B) Rational of Premo Autophagy Tandem Sensor RFP‐GFP‐LC3B kit for monitoring autophagic flux. By combining an acid‐sensitive GFP with an acid‐insensitive RFP, the change from autophagosome (neutral pH) to autolysosome (with an acid pH) can be visualized by imaging the specific loss of the GFP fluorescence, leaving only red fluorescence. Image shows in yellow autophagosomesand in red autolysosomes. C) Puncta of autophagosomes (yellow) and autolysosomes (red) per cell were measured using ImageJ software. The mean value ± s.d. of at least five cells is shown on the plots (*n* = 3). The results were presented as means ± standard deviation; values were compared to CTR by one‐way ANOVA following Tukey test, *p < 0.0332, **p < 0.0021, ***p < 0.0002 in comparison to CTR; ^#^ *p* < 0.0332, ^##^ *p* < 0.0021, ^###^ *p* < 0.0002 in comparison to NH_4_Cl 0.5 × 10^−3^ m. Image correlation analysis was performed through ImageJ Jacop. PCC = Pearson's correlation coefficient. −1 < PCC < 0 = negative correlation; PCC = 0 no correlation; 0 < PCC ≤ 1 = positive correlation. The mean correlation coefficient value ± s.d. of *n* = 3 images is shown on the merge images.

In addition to immunoblotting, an alternative approach to assess autophagy is by immunofluorescence. For this, we studied autophagic flux in Huh7cells transfected with Premo Autophagy Tandem Sensor RFP‐GFP‐LC3B Kit. Huh7 cells were treated with NH_4_Cl 0.5 × 10^−3^ m in the presence or absence of c‐Myc I for 48 h and then analyzed by confocal microscope (**Figure** **4**A). The tandem sensor RFP‐GFP‐LC3B allows for visualization of the transition from autophagosome to autolysosome through combining acid‐sensitive GFP with acid‐insensitive RFP. The specific loss of GFP fluorescence indicates acidification of the autophagosome after lysosomal fusion. When autophagy is activated, the Premo Tandem Autophagy Sensor labels the punctate autophagosomes, which show positive signals for both GFP and RFP. After the lysosome merges, the pH decreases, which quenches the GFP, resulting in autolysosomes becoming red (a schematic representation of the mechanisms is illustrated in Figure 4B)

As shown in Figure 4A, the treatment with low concentration of ammonia (NH_4_Cl 0.5 × 10^−3^ m) induced a statistically significant increase in the number of yellow (GFP+RFP+) and red (GFP‐RFP+) puncta compared to CTR cells (Figure 4A,B). Instead, Huh7 cells treated with c‐Myc I displayed a reduction in the number of yellow (GFP+RFP+) and red (GFP‐RFP+) puncta if compared with Huh7 cells treated with NH_4_Cl 0.5 × 10^−3^ m alone (Figure 4A,B), thus suggesting an inhibition in the autophagic flux at the early stage. Indeed, as expected, the inhibition of c‐Myc in Huh7 cells treated with NH_4_Cl 0.5 × 10^−3^ m induced a reduction in yellow (GFP+RFP+) and red (GFP‐RFP+) puncta with respect to cells treated with NH_4_Cl 0.5 × 10^−3^ m alone (Figure 4A,B), thus suggesting a reduction in autophagy flux.

To verify that the observed phenomenon was not due to an off‐target effect of the c‐Myc inhibitor, we confirmed that LC3‐II levels were also decreased after the knocked down of c‐Myc (sh‐c‐Myc). Indeed, the evaluation of autophagy markers in Huh7 sh‐Myc cells showed decreased LC3II and p62 after knockdown of c‐Myc, confirming that c‐Myc knockdown is involved in autophagy inhibition (Figure S3A,B, Supporting Information).

Moreover, we further confirmed the involvement of c‐Myc in autophagy regulation through c‐Myc overexpression in Huh7 cells treated with NH_4_Cl 5 × 10^−3^ m. Indeed, overexpression of c‐Myc in Huh7 cells reverses the expression of autophagy markers. In details, SQSTM1/P62 and LC3II reduced expression levels were found after the overexpression of c‐Myc, although there was the presence of high ammonia concentrations, thus suggesting autophagy activation (Figure S4A,B, Supporting Information).

Altogether, these data suggested that c‐Myc is important for the ammonia‐induced activation of autophagy in Huh7 cells.

### 2.3 Molecular Mechanisms Underlying c‐Myc Autophagy Modulation under Different Ammonia Concentrations

To further investigate the molecular mechanism involved in ammonia modulation of autophagy, we examined potential molecular players upstream of c‐Myc, like β‐catenin and of its best regulator GSK3β under both high and low concentrations of ammonia.

**Figure 5:**
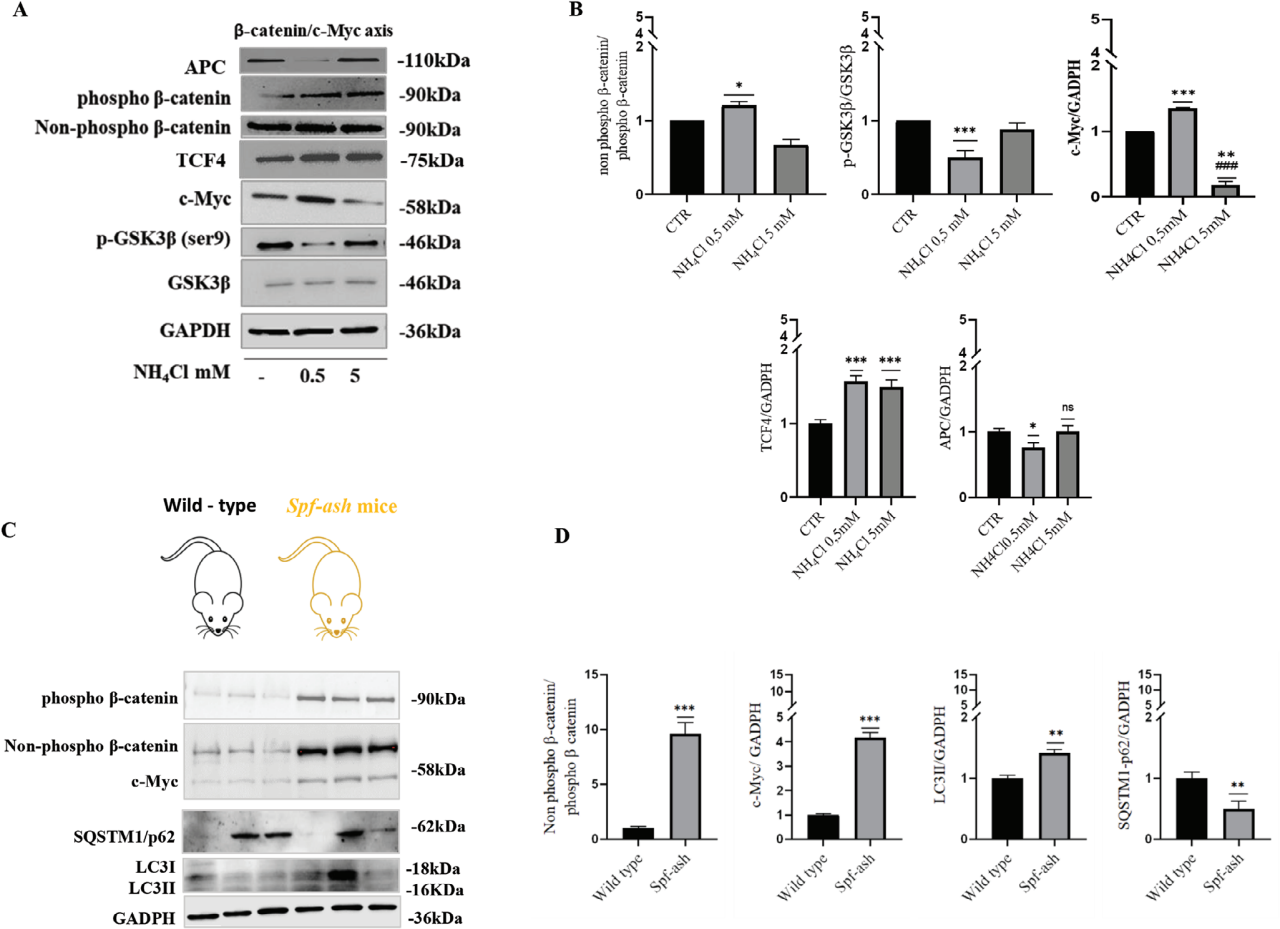
A,B) Western blot analyses and densitometric quantification of non‐phospho‐β‐catenin, phospho‐β‐catenin, c‐Myc, phospho‐GSK3β (ser9), GSK3β, APC, TCF4 in Huh7 cells after 48 h of treatment with NH_4_Cl 0.5 × 10^−3^ m and 5 × 10^−3^ m. GADPH is used as loading control (*n* = 3). The results were presented as means ± standard deviation; values were compared to CTR by one‐way ANOVA following Tukey test, *p < 0.0332, **p < 0.0021, ***p < 0.0002 in comparison to CTR; ^#^ *p* < 0.0332, ^##^ *p* < 0.0021, ^###^ *p* < 0.0002 in comparison to NH_4_Cl 0.5 × 10^−3^ m; ^+^ *p* < 0.0332, ^++^ *p* < 0.0021, ^+++^ *p* < 0.0002 in comparison to NH_4_Cl 5 × 10^−3^ m. C,D) Western blotting and densitometric analysis of phospho‐β‐catenin, non‐phospho β‐catenin, c‐Myc and of the autophagy markers SQSTM1/p62, LC3 in liver of wild type or *spf‐ash* mice (*n* = 3). The results were presented as means ± standard deviation; values were compared to CTR through Student's *t*‐test, *p < 0.05, **p < 0.01.

An increase in the non‐phospho‐β‐catenin/ phospho‐β‐catenin protein ratio was detected in Huh7 cells treated with 0.5 × 10^−3^ m NH_4_Cl but not under high concentrations (NH_4_Cl 5 × 10^−3^ m) (**Figure** **5**A,B). Regulation of β‐catenin occurs primarily by stabilization through the Wnt signaling pathway, which phosphorylates and inactivates GSK3β, the canonical inhibitor of β‐catenin, that induces β‐catenin proteasomal degradation^[^ ^33^ ^]^ through its phosphorylation. The reduced p‐GSK3β/GSK3β protein ratio found, under low ammonia concentrations and not under high, suggested that β‐catenin reduced activity occurred in a GSK3B independent way in both conditions.

The main mechanism for controlling β‐catenin activity is on the level of protein degradation, but β‐catenin nuclear localization and hence its transcriptional activity may additionally be regulated via nuclear import carried out by TCF4 and BCL9 and via nuclear export by APC and axin.^[^ ^34^ ^]^ This prompted us to investigate the effect of NH_4_Cl on TCF4 and APC expression levels. APC and TCF4 modulation are important for β‐catenin nuclear retention and thus for its transcriptional activity. We found that Huh7 cells treated with NH_4_Cl 0.5 × 10^−3^ m displayed a reduction in APC expression and an increase in TCF4 expression, in agreement with the increase in the active dephosphorylated form of β‐catenin and its target c‐Myc. On the contrary, no statistically significant variations were found in APC expression after NH_4_Cl 5 × 10^−3^ m treatment but only an increase in TCF4 expression. Thus, these findings collectively suggest that ammonia‐induced autophagy, mediated by c‐Myc, relies on activated β‐catenin. While β‐catenin activity under high ammonia concentrations is independent of the GSK3‐β pathway, APC, and TCF4, the observed increase in TCF4 levels and decrease in APC expression under low ammonia conditions may enhance β‐catenin's transcriptional activity.

The modulation of the β‐catenin/c‐Myc axis was further investigated in a *spf‐ash* mice, a genetic model of OTC (ornithine‐transcarbamylase enzyme) deficiency and an in vivo model of hyperammonemia. Consistent with the data in Huh7 cells treated with low concentrations of NH_4_Cl, in livers of *spf‐ash* mice we observed increased expression of c‐Myc and active dephosphorylated form of β‐catenin compared to WT control mice (Figure 5C,D). Phosphorylated β‐catenin was also found to be increased. Reduced expression of p62/SQSTM1 and increased LC3II in *spf‐ash* mice compared to WT mice suggested activation of hepatic autophagy (Figure 5C,D). These data suggest that also in vivo ammonia can modulate β‐catenin/c‐Myc axis and that this could be involved in the modulation of autophagy, but further studies are needed.

### 2.4 β‐Catenin/c‐Myc Axis Supports a Cytoprotective Autophagic Response to Stress Induced by Ammonia

To investigate the functional role of β‐catenin/c‐Myc axis modulation in Huh7 cells exposed to low and high ammonia concentrations, cell viability was evaluated using the LIVE/DEAD vitality assay. This fluorescence‐based method distinguishes between dead cells (green‐stained) and living cells (blue‐stained).

Initially, cells were treated with 0.5 and 5 × 10^−3^ m NH_4_Cl, revealing a reduction in cell viability under both conditions. This decrease was more pronounced at higher ammonia concentration when the β‐catenin/c‐Myc axis and autophagy were inhibited.

**Figure 6:**
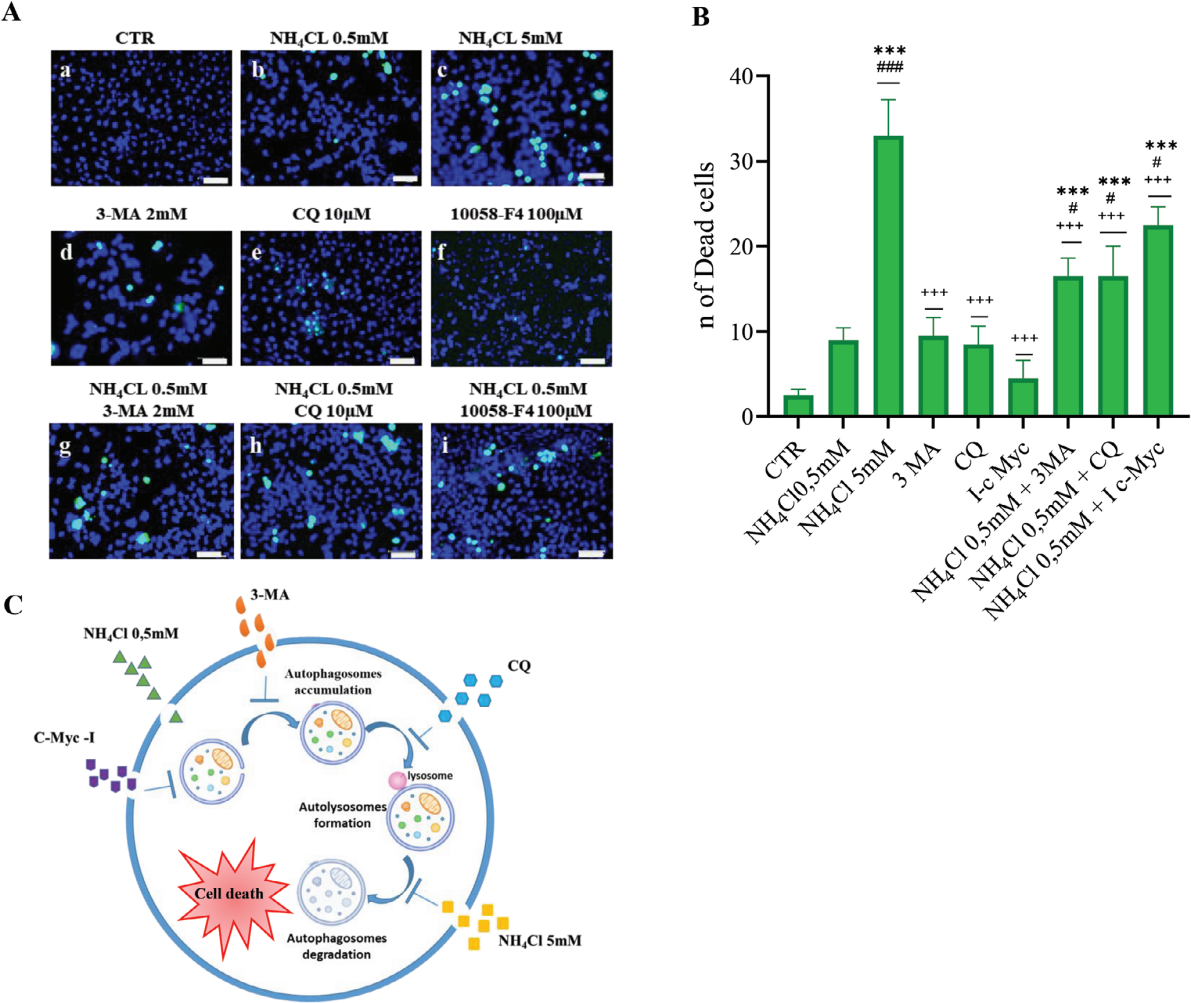
A (a–i) Huh7 cells were cultured in presence or absence of the indicated drugs (NH_4_Cl 0.5 × 10^−3^ m; 3‐methyladenine 2 × 10^−3^ m; chloroquine 10 × 10^−6^ m, 10058‐F4 100 × 10^−6^ m) for 24 h and then stained with the LIVE/DEAD kit and observed at the fluorescence microscope. Image shows in blue live cells, and in green dead cells. Scale bar is 100 µm. B) Live/dead cell viability quantification: number of dead cells was calculated using ImageJ, data shown are mean ± SD, *n* = 3. The results were presented as means ± standard deviation; values were compared to CTR by one‐way ANOVA following Tukey test, *p < 0.0332, **p < 0.0021, ***p < 0.0002 in comparison to CTR; ^#^ *p* < 0.0332, ^##^ *p* < 0.0021, ^###^ *p* < 0.0002 in comparison to NH_4_Cl 0.5 × 10^−3^ m; ^+^ *p* < 0.0332, ^++^ *p* < 0.0021, ^+++^ *p* < 0.0002 in comparison to NH_4_Cl 5 × 10^−3^ m. C) Illustration of autophagy inhibitors mechanisms of action: 3MA (3‐methyladenine) inhibits autophagosomes maturation by inhibiting phosphatidylinositol‐4,5‐bisphosphate 3‐kinase (PI3K) activity, while CQ (chloroquine) could block autolysosomes fusion, instead c‐Myc inhibition impairs autophagosomes formation, while NH4Cl 5 × 10^−3^ m blocks autophagosomes degradation.

Thus, to demonstrate the cytoprotective role of this axis, we treated Huh7 cells with NH_4_Cl 0.5 × 10^−3^ m in combination with three different inhibitors: 3MA (3‐methiladenine) and CQ (Chloroquine), two well‐known autophagy inhibitors, or c‐Myc I (10058‐F4) as illustrated in **Figure** **6**C. 3MA inhibits autophagosomes maturation by inhibiting phosphatidylinositol‐4,5‐bisphosphate 3‐kinase (PI3K) activity, while CQ could block autolysosomes fusion, instead c‐Myc inhibition impairs autophagosomes formation. As shown in Figure 6A,B, the inhibition of autophagy through 3MA, CQ, or c‐Myc I in cells treated with NH_4_Cl 0.5 × 10^−3^ m caused an increase in the number of dead cells with respect to Huh7 cells treated with NH_4_Cl 0.5 × 10^−3^ m alone.

Specifically, the number of dead cells was higher in Huh7 cells treated with 0.5 × 10^−3^ m NH_4_Cl combined with the c‐Myc inhibitor, similar to the effect observed under high ammonia concentrations.

These data suggested how the activation of the β‐catenin/c‐Myc axis, supporting the autophagy process, is important to counteract ammonia cytotoxicity.

## 3 Discussion

Ammonia, a key product of nitrogen metabolism, is used in various metabolic reactions but is mainly a waste product that is quickly converted by the healthy liver into less harmful compounds such as glutamine and urea.^[^ ^17^ ^]^

Normal levels of ammonia in the blood range from 0 to 50 × 10^−6^ m for healthy adults, 0 to 150 × 10^−6^ m for infants, while it can reach up to 1 × 10^−3^ m for individuals with hyperammonemia.^[^ ^17^ ^]^ High levels of ammonia that exceed normal levels are harmful to neurons and occasionally to other types of cells as well.

Previous research discovered that ammonia triggers autophagy in cultured cancer cells.^[^ ^3^, ^4^ ^]^ Autophagy is a recycling process that is well‐preserved and is involved in the degradation of cytoplasmic components and organelles in the lysosomes. It has a significant impact during various stress conditions like lack of nutrients or growth factors, oxidative stress, and accumulation of damaged organelles.^[^ ^35^ ^]^ The relationship between ammonia levels and autophagy is particularly interesting, as it can provide insights into cellular responses to metabolic disturbances.

In this paper, the selected concentrations of NH_4_Cl (0.5–5 × 10^−3^ m) are chosen to reflect a range of hyperammonemia severity seen in clinical settings.^[^ ^17^ ^]^ In particular, 0.5 × 10^−3^ m NH_4_Cl is on the lower end of the spectrum for experimentally modeling hyperammonemia. It represents a mild to moderate increase in ammonia levels, which is within the range observed in some clinical hyperammonemia cases. At this concentration, ammonia is known to active autophagy.^[^ ^4^ ^]^ Instead, 5 × 10^−3^ m NH_4_Cl is significantly higher and represents severe hyperammonemia, which is seen in more acute or severe clinical cases. Notably, such high ammonia concentrations, like 5 × 10^−3^ m, have been reported to inhibit autophagy.^[^ ^15^, ^16^ ^]^

Low ammonia levels are generally indicative of a lower level of metabolic stress or toxicity. Activating autophagy in this context can help maintain cellular health by removing damaged organelles, misfolded proteins, and other cellular debris. It serves as a preventative measure, ensuring that cells remain in optimal condition by keeping potential sources of stress in check. With lower ammonia levels, cells might be in a better state to engage in autophagy without the immediate threat of toxicity. This activation can support regular cellular turnover and repair processes, aiding in the overall maintenance of cellular function and longevity. High ammonia levels typically indicate a state of metabolic imbalance or increased toxicity, such as in conditions like liver dysfunction or urea cycle disorders.^[^ ^11^, ^12^, ^13^, ^14^ ^]^ In such scenarios, the accumulation of ammonia can contribute to oxidative stress and disrupt cellular homeostasis. Blocking autophagy during high ammonia levels could be a cellular response to prevent further degradation of essential cellular components when the cell is already under significant stress. However, when ammonia levels are high, blocking autophagy can exacerbate cellular damage. Since autophagy plays a crucial role in removing damaged organelles and proteins, inhibiting this process might lead to the accumulation of dysfunctional cellular components. This can further impair cellular function and contribute to the progression of metabolic disorders or cellular toxicity.^[^ ^17^, ^36^ ^]^ Thus, the activation of autophagy with low ammonia levels supports cellular maintenance and detoxification, while the blockage of autophagy with high ammonia levels might be a protective measure against excessive damage, though it could also lead to the accumulation of cellular debris and increased damage if the high ammonia persists. Understanding these dynamics can be crucial for managing conditions related to ammonia metabolism and cellular stress. However, the mechanism by which ammonia induces or inhibits the autophagy process remains controversial. From literature research comes out that ammonia induces autophagy in a Ulk1/2 dependent pathway but is independent of mTORC1,^[^ ^4^ ^]^ or in a mTORC1 and ULK1 independent mechanisms requiring ATG5.^[^ ^3^ ^]^ The differences in mTORC1 responses to ammonia are unknown and they could depend on cell and tissue contexts.^[^ ^8^ ^]^

In this work, we found that ammonia can modulate the autophagy process through the regulation of c‐Myc expression, in a cellular model of hepatocellular carcinoma (Huh7). Indeed, we showed that when Huh7 cells displayed elevated levels of c‐Myc, under low ammonia concentrations, the autophagy process is active, instead it is inhibited under high ammonia concentrations when c‐Myc expression was reduced. In agreement with these observations, c‐Myc inhibition with the small molecule inhibitor 10058‐F4, in cells treated with NH_4_Cl 0.5 × 10^−3^ m, resulted in impaired autophagy. On the contrary, c‐Myc overexpression in Huh7 cells treated with NH_4_Cl 5 × 10^−3^ m reverse the expression of autophagy markers.

The involvement of c‐Myc in the regulation of the autophagy process was demonstrated for the first time by Toh and colleagues that proved how siRNA mediated knockdown of c‐Myc inhibited autophagy, in human embryonic renal (HEK293) cells thus resulting in the accumulation of the autophagy substrate p62/SQSTM1.^[^ ^23^ ^]^ Toh and colleagues^[^ ^23^ ^]^ identified JNK1 and Bcl2 phosphorylation as plausible effectors involved in the regulation of autophagy by Myc suppression, and that this is likely mediated through changes in downstream Bcl2 Beclin1 interactions. Elucidation of the molecular mechanisms involved in Myc regulation of autophagy is important for understanding the feasibility and the potential side effects that could arise from the inactivation of Myc and its affected downstream targets like mTOR, AMPK, p53, Nf‐KB, and Bcl2 family proteins. This is of interest and importance for intervention in ammonia related disorders such as hyperammonemia and tumors. However, in this paper we focused our attention on c‐Myc as a target of the Wnt/β‐catenin pathway known to be involved in the zonal regulation of ammonia metabolizing enzymes in the adult liver^[^ ^18^, ^19^ ^]^ and in the regulation of autophagy process.

The Wnt signaling pathway and its interaction with the β‐catenin/c‐Myc axis is a critical area of research in understanding cellular behavior, particularly in the context of self‐renewal, differentiation, and autophagy. The Wnt pathway is a fundamental signaling cascade that regulates various cellular processes, including proliferation, differentiation, and apoptosis.^[^ ^37^, ^39^ ^]^ In its canonical pathway, Wnt ligands bind to receptors (Frizzled and LRP5/6), leading to the stabilization and nuclear translocation of β‐catenin. Within the nucleus, β‐catenin interacts with TCF/LEF transcription factors to activate target genes, including c‐Myc, which is a well‐known regulator of cell cycle progression, growth and metabolism.^[^ ^20^ ^]^ The β‐catenin/c‐Myc axis could influence autophagic pathways by adapting metabolic flux to meet energy demands, thus influencing autophagy or by activating or repressing genes linked to lysosome formation and autophagy based on ammonia levels.^[^ ^21^, ^22^, ^38^ ^]^ Understanding how cells adapt to fluctuating ammonia concentrations via Wnt signaling is crucial for developing interventions for metabolic disorders and cancer. Thus, exploring the crosstalk between Wnt signaling, the β‐catenin/c‐Myc axis, and autophagy under varying environmental conditions, like ammonia concentration, provides a foundation for understanding how cellular homeostasis is maintained.^[^ ^35^, ^40^ ^]^ This knowledge has the potential for the development of therapeutic approaches to manipulate autophagy and address diseases linked to metabolic dysregulation and stress adaptation.

Here, we evaluated whether ammonia perturbs autophagy flux through the β‐catenin/c‐Myc axis. Interestingly, we found an increase in the active dephosphorylated form of β‐catenin in cells treated with NH_4_Cl 0.5 × 10^−3^ m where the expression of c‐Myc was increased but not in Huh7 cells treated with NH_4_Cl 5 × 10^−3^ m. We went on to better elucidate the molecular mechanism involved in ammonia‐induced regulation of autophagy through c‐Myc. In details, we analyzed the expression of GSK3β which is the best‐recognized regulator of β‐catenin. GSK3β is activated by dephosphorylation and inactivated by phosphorylation on Ser9 site, with consequent changes in β‐catenin stabilization and transcriptional activity.^[^ ^33^ ^]^ Indeed, phosphorylated β‐catenin is rapidly degraded via proteasome mediated proteolysis. Interestingly, the reduced phosphorylation status of GSK3β found, under low but not under high ammonia concentrations, suggested how in these conditions β‐catenin degradation occurs in a GSK3β independent way. Although the regulation of β‐catenin activity is thought to occur mainly on the level of protein phosphorylation and degradation^[^ ^41^, ^42^, ^43^, ^44^ ^]^ its nuclear uptake and/or retention may additionally be regulated by multiple signaling interactors, such as TCF4, BCL9, adenomatous polyposis coli (APC), and axis inhibition protein 2 or conductin (Axin2).^[^ ^34^ ^]^ Our results show a reduction in APC expression and an increase in TCF4 expression in cells treated with NH_4_Cl 0.5 × 10^−3^ m but no variations in APC expression when cells were treated with NH_4_Cl 5 × 10^−3^ m. Thus, according to our data, in Huh7 cells treated with NH_4_Cl 0.5 × 10^−3^ m, although β‐catenin stabilization and its transcriptional activity occurred independently of GSK3‐β pathway, its transcriptional activity could be regulated by TCF4 and APC. Instead, these modifications are lost under high ammonia concentrations, probably due to ammonia negative effect on cell functions, signal transduction, as well as on the phosphorylation status of some proteins as a consequence of changes in pH or membrane potential.^[^ ^45^, ^46^, ^47^ ^]^ The noncanonical degradation of β‐catenin during hyperammonemia was also observed in HEK cells where the degradation of β‐catenin during hyperammonemia occurs through a mechanism IKK dependent but GSK3β independent.^[^ ^48^ ^]^

In line with these cellular data, we found that *spf‐ash* mice, a model of constitutive hyperammonemia,^[^ ^16^, ^49^ ^]^ displayed elevated levels of c‐Myc and the active dephosphorylated form of β‐catenin. These modifications were associated with the activation of the autophagy process in *spf‐ash* mice with respect to WT mice. These data suggested that in vivo ammonia can also modulate β‐catenin/c‐Myc axis and that this axis could be involved in the modulation of autophagy, but further studies are needed. According to Soria et al., the activation of the autophagy process suggested the initiation of a compensatory, pro‐survival mechanism, important in the detoxification of ammonia.^[^ ^49^, ^50^ ^]^ Thus, we verified whether the induction of autophagy by β‐catenin/c‐Myc axis was a cytoprotective response to stress induced by ammonia. Autophagy is largely believed to function as pro‐survival process due to its critical role in cellular energy and nutrition homeostasis, indeed autophagy inhibition compromise cell viability. The LIVE/DEAD vitality assay demonstrated that the inhibition of autophagy through 3MA, CQ, or c‐Myc I in cells treated with NH_4_Cl 0.5 × 10^−3^ m reduced cell vitality with respect to controls.

In particular, the reduction in cell viability was higher in all cases characterized by inhibition of autophagy, but in particular after the inhibition of c‐Myc in cells treated with NH_4_Cl 0.5 × 10^−3^ m, thus showing how the activation of this axis supports a cytoprotective autophagy response.

Thus, in summary, from this study, it emerged that the β‐catenin/c‐Myc axis has an important role in the regulation of autophagy by ammonia. Moreover, the activation of autophagy through the β‐catenin/c‐Myc axis could have a protective role both in vitro and in vivo against the toxic effects of ammonia. Being the actual therapeutic options to treat hyperammonemia unsatisfactory,^[^ ^49^, ^51^ ^]^ the discovery of the β‐catenin/c‐Myc axis involvement in autophagy regulation by ammonia opens new landscapes for the development of novel therapeutic targets and novel treatment options.

## 4 Experimental Section

### Cell Culture and Reagents

The human hepatocellular carcinoma cell line (Huh7) was purchased from the American Type Culture Collection (ATCC) and cultured in low glucose Dulbecco's modified Eagle medium (DMEM) (SIGMA) supplemented with 10% (v/v) fetal bovine serum (FBS) (BIOWEST), 1% (v/v) l‐glutamine (SIGMA), 1% (v/v) penicillin/streptomycin (SIGMA) in a humidified incubator at 37 °C in an atmosphere of 5% CO_2_.

Cells were seeded at a density of 5 × 10^4^ cells/well.

All chemicals used in this study were dissolved in dimethyl sulfoxide (DMSO) except for NH_4_Cl which was suspended in water. The following chemical compounds were used for the treatment at the following concentration: NH_4_Cl (0.5/5 × 10^−3^ m) (Sigma); Bafilomycin A1 (100 × 10^−9^ m) (BioViotica); 10058‐F4 (100 × 10^−6^ m) (Selleckchem); Rapamycin 1 × 10^−6^ m (Selleckchem); 3‐methyladenine 2 × 10^−3^ m (Selleckchem); chloroquine 10 × 10^−6^ m (Sigma).

### Live/Dead Cell Assay

Cells were seeded at a density of 10 × 10^4^ cells/well and cultured for 24 h with NH_4_Cl 0.5 × 10^−3^ m and pretreated for 1 h with or without 3‐MA 2 × 10^−3^ m, chloroquine 10 × 10^−6^ m, or 10058F4 100 × 10^−6^ m. At the end of the treatment, cells were stained with the Live/Dead cell kit (Invitrogen) according to the manufacturer's instructions and observed at the fluorescence microscope. live cells are shown in blue, while dead cells are shown in green. Dead cells were quantified using ImageJ.

### Immunoblotting

The cells were lysed with RIPA buffer (Cell Signaling technology). After high‐speed centrifugation of the cell lysates, the protein concentrations of the supernatants were determined using the Bradford assay (Bio‐Rad, Hercules, CA). The proteins were separated on sodium dodecyl sulfate‐polyacrylamide (SDS‐PAGE) gels and transferred to nitrocellulose film except for the detection of LC3 protein, for which PVDF film was used. The membranes were blocked in BlottoA (Santa Cruz Biotechnology, Inc.), which is composed of 5% milk, 0.05% Tween‐20 for 1 h under agitation and subsequently incubated with primary antibodies for 1.5 hour, followed by incubation with secondary antibodies for 1 h at room temperature. The blots were developed using a horseradish peroxidase (HRP) chemiluminescent substrate reagent kit (GE Healthcare). The following primary antibodies were used (1:1000) and were purchased by Cell Signaling Technology: anti‐SQSTM1/p62, anti‐LC3A/B, anti‐c‐Myc D3N8F, anti‐phospho‐β‐catenin (Ser33/37/Thr41), anti‐non‐phospho(active) β‐catenin (Ser33/37/Thr41) (D13A1), anti‐GSK3β(D5C5Z) XP, anti‐phospho‐GSK3β (Ser9), anti‐TCF4, anti‐APC, anti‐CyclinD1, anti‐α‐tubulin, anti‐actin, and anti‐GADPH. Anti‐Beclin‐1 was purchased by Calbiochem. GADPH, α‐tubulin, and actin were used as loading control. The following secondary antibodies were used (1:2000): anti‐rabbit IgG, HRP‐link antibody (Cell Signaling Technology); anti‐mouse IgG, HRP‐link antibody (Cell Signaling Technology).

### Immunofluorescence

Cells were stained with Lysotracker Red DND‐99 50 × 10^−9^ m (Invitrogen) according to the manufacturer's instructions. After staining with Lysotracker red, cells were washed with RPMI without phenol red and then incubated with Monodansylcadaverine 0.05 × 10^−3^ m in RPMI without phenol red at 37 °C for 10 min. At the end, cells were washed and assayed by a confocal microscope.

Immunofluorescence images were acquired with confocal scanner laser microscopy (CSLM) using a Leica TCS SP8 Confocal microscope. Images were acquired with the LasAF software using a 63× oil‐immersion objective (HC PL APO CS2 63×/1.40 OIL, Leica Germany) or 100× oil‐immersion objective. The pinhole was set at 1 Airy unit. Monodansylcadaverine fluorescence was observed using a 405 nm continuous diode laser and fluorescence emission was detected in the spectral window, between 420 and 500 nm by a transmission photomultiplier tube (PMT). Lysotracker red fluorescence was observed using a 570 nm argon‐ion laser for fluorophore excitation, while emission was detected in a spectral window between 577 and 590 nm by a transmission PMT. The power of each laser line and the gain were identical for each experiment to ensure the comparability of the images.

Image correlation analysis was performed through ImageJ Jacop. PCC = Pearson's correlation coefficient. −1 < PCC < 0 = negative correlation; PCC = 0 no correlation; 0 < PCC ≤ 1 = positive correlation. The mean correlation coefficient value ± s.d. of *n* = 3 images is shown on the merge images.

### Analysis of Autophagosome and Autolysosome Formation

To analyze the autophagic flux in Huh7 cells, the commercially available Premo Autophagy Tandem Sensor RFP‐GFP‐LC3B Kit (Invitrogen, Carlsbad, CA, USA) was used. Huh7 cells were transduced with BacMam reagents while in suspension in complete cell medium and then plated in a six‐well plate at a density of 4 × 10^4^ cells/well and incubated overnight (16 h).

After 16 h of incubation with BacMam RFP‐GFP‐LC3B particles, Huh7 cells were treated with NH_4_Cl 0.5 × 10^−3^ m and/or c‐Myc inhibitor (100 × 10^−6^ m) for 48 h and then visualized using confocal laser scanning microscopy. The neutral pH autophagosome, identified by GFP emission, and acidic autolysosome, identified by RFP emission, were visualized using standard FITC/TRITC filter sets.

### Mouse Procedures

Mouse procedures were performed in accordance with regulations and were authorized by the Italian Ministry of Health. Details about mice treatments were previously described.^[^ ^50^ ^]^ Briefly, spf‐ash mice (B6EiC3Sn a/A‐OTCSpf‐Ash/J; Jackson Laboratories) were housed in individually ventilated cages, maintaining a temperature of 22 °C (±2 °C), relative humidity of 55% (±10%), 15–20 air exchanges per hour, and 12‐h light/12‐h dark cycle and receiving a standard chow diet and water ad libitum. Wild‐type littermates were used as controls. 12‐week‐old male mice were sacrificed by cervical dislocation, and liver samples were harvested for analyses.

### Statistical Analysis

The results are presented as means ± standard deviation from at least three biological replicates. For multiple comparisons, data were analyzed by using the one‐way or two‐way analysis of variance (ANOVA) with post hoc Tukey test. Statistical analyses were performed, and a value of *p* < 0.0332 was accepted as the level of significance (GraphPad Prism 8.0). The following statistical significance representations were used: *p < 0.0332, **p < 0.0021, ***p < 0.0002. Instead, to test for statistical differences between two conditions, an unpaired Student's *t*‐test was used. Significance levels were considered as following: ***p < 0001, **p < 0.01, *p ≤ 0.05.

### Ethics Approval Statement

Mouse procedures were performed in accordance with regulations and were authorized by the Italian Ministry of Health. Details about mice treatments were previously described.^[^ ^50^ ^]^

## Conflict of Interest

The authors declare no conflict of interest.

## Supporting information



Supporting Information

## Data Availability

The data that support the findings of this study are available from the corresponding author upon reasonable request.
