# Supplementary material for: β‐Catenin/c‐Myc Axis Modulates Autophagy Response to Different Ammonia Concentrations
Source: Adv Biol (Weinh). 2025 Jan 11;9(3):2400408. doi: 10.1002/adbi.202400408 (PMC11911958; doi:10.1002/adbi.202400408)
Supplement: Supplementary file 1 — Supporting Information [file ADBI-9-2400408-s001.pdf]

ADVANCED BIOLOGY

Supporting Information

for *Adv. Biology*, DOI 10.1002/adbi.202400408

β-Catenin/c-Myc Axis Modulates Autophagy Response to Different Ammonia Concentrations

S. Sergio, B. Spedicato, G. Corallo, A. Inguscio, M. Greco, D. Musarò, D. Vergara, A. F. Muro, G. De Sabbata, L. R. Soria, N. Brunetti Pierri and M. Maffia**

Fig.S1 uncropped scan Fig. 1B

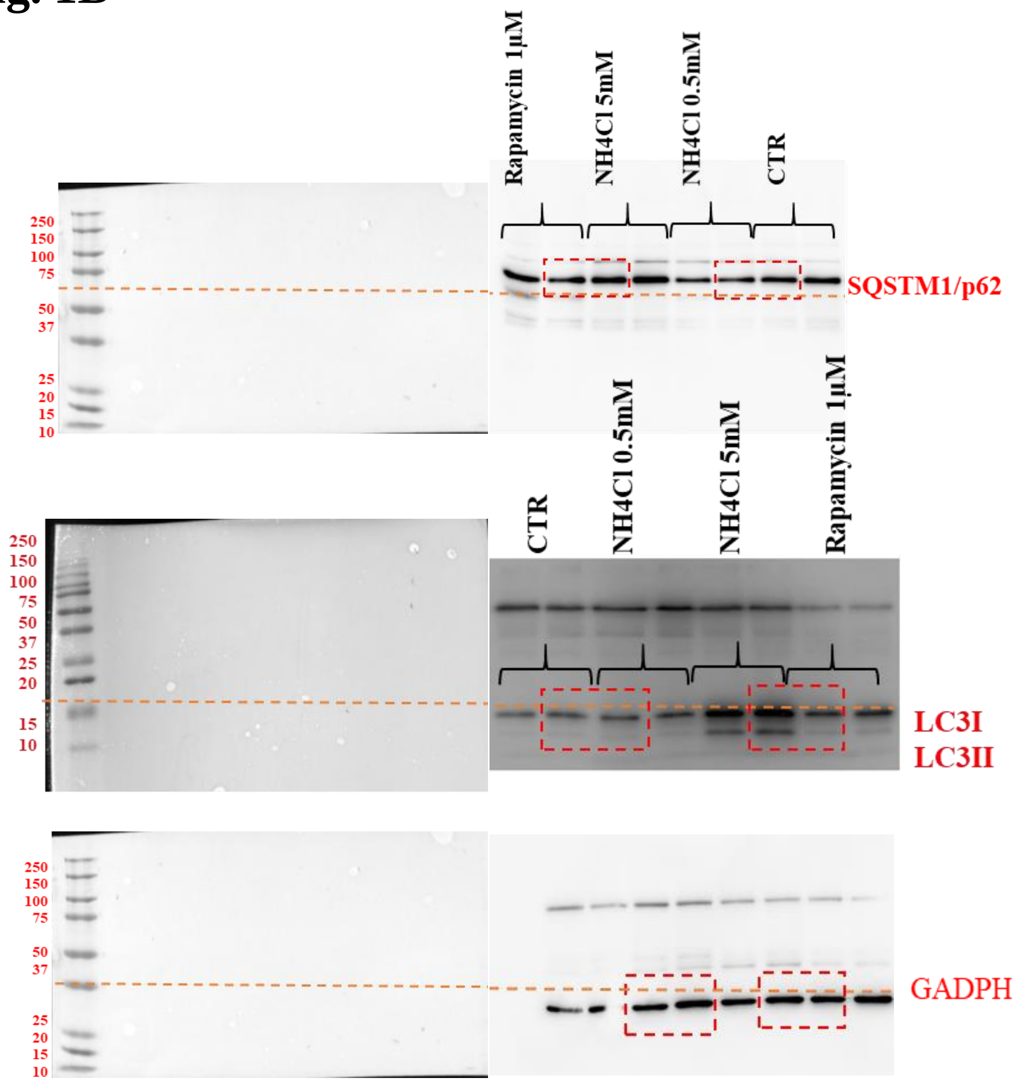

Fig. S1: uncropped scan showing expression levels of autophagy markers SQSTM1/p62,and LC3 in Huh7 cells, after 48h of treatment with NH_4Cl 0.5/5 mM and Rapamycin 1µM. GADPH is used as loading control. Samples for the detection of SQSTM1/p62, LC3II were run on the same gel but were not contiguous.

Fig.S2

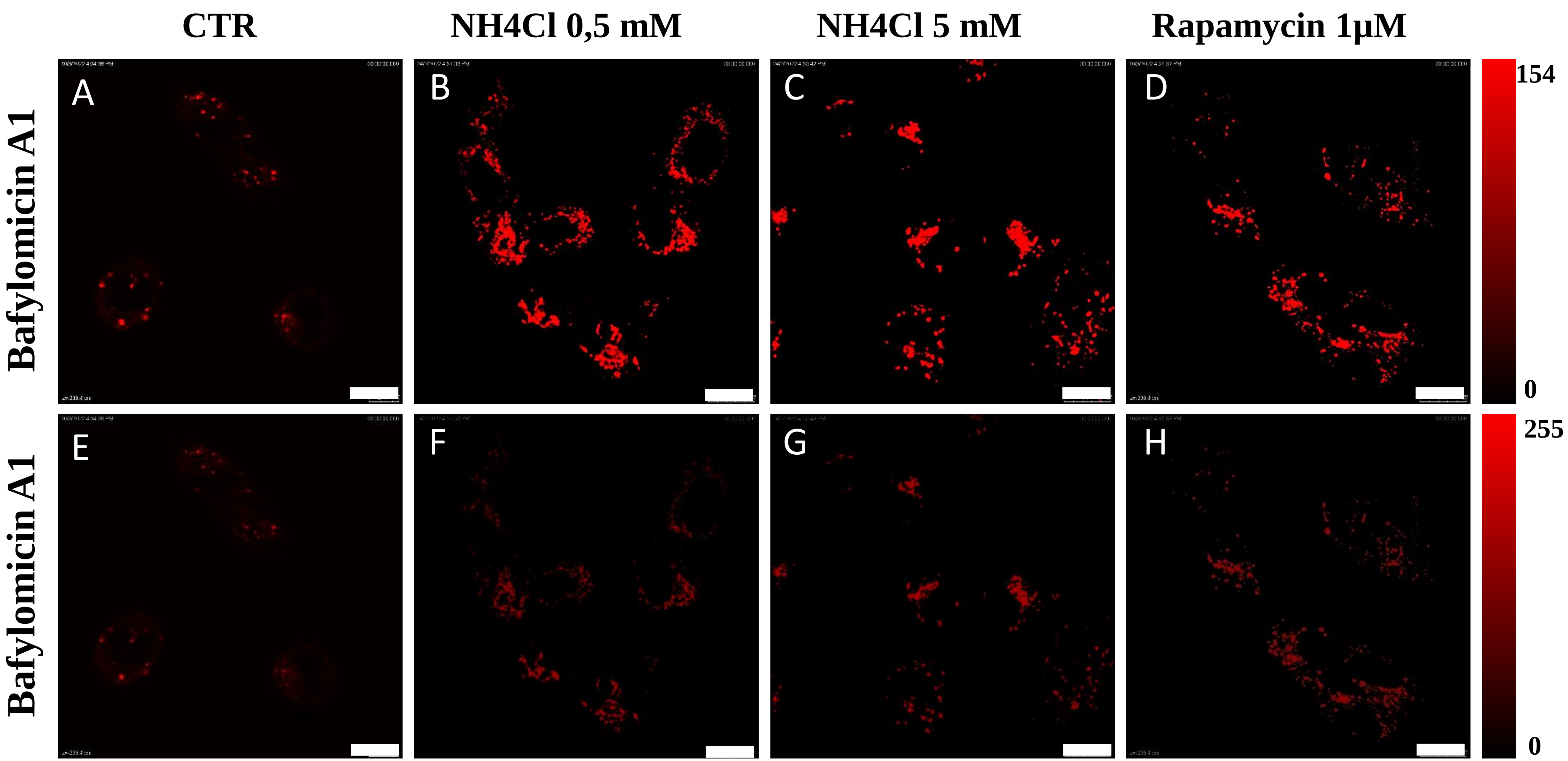

Fig.S2: Huh7 cells were treated for 48h with the indicated concentration of ammonia and rapamycin. Bafilomycin A1 (100nM) was added 4h before to perform the staining with mondansylcadaverine for autophagosomes (in blue) and lysotraker red for lysosomes (in red). Scale bar is 10 µm. In panel A-D color scale has a maximum value of 154 A.U. against the 255 A.U., used for original images (E-H), to better display Lysotraker red fluorescence signal, that could be affected by Bafilomycin A1 treatment.

Fig.S3

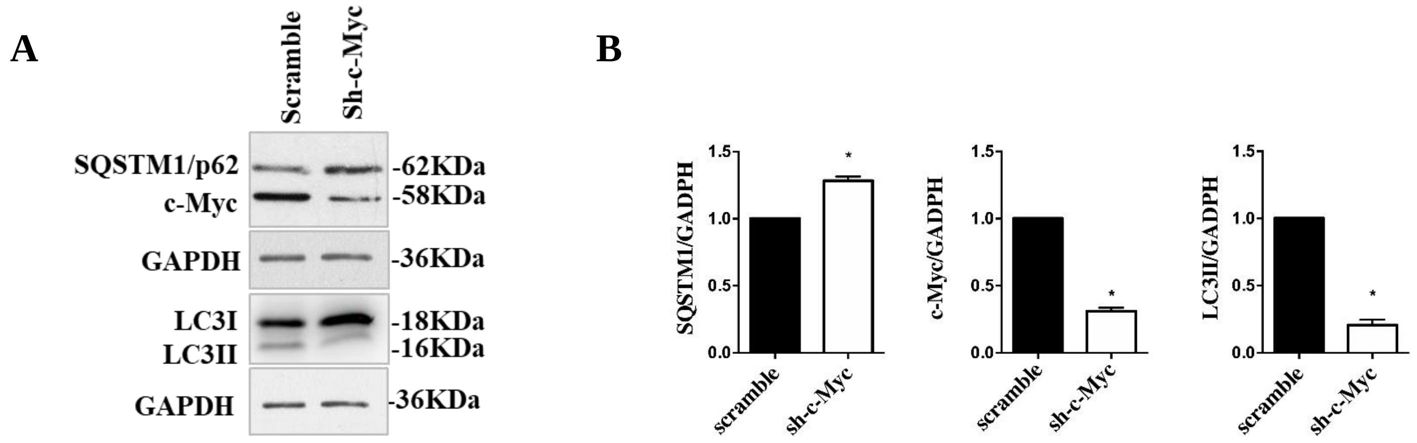

Fig.S3 A,B) Western blot analyses and densitometric quantification of SQSTM1/p62, LC3II, c-Myc in Huh7 cells subjected to Control shRNA Lentiviral Particles-A or c-Myc shRNA Lentiviral particles-A . GADPH is used as loading control. (n=3)*P<0,05, ** P<0,01

Fig.S4

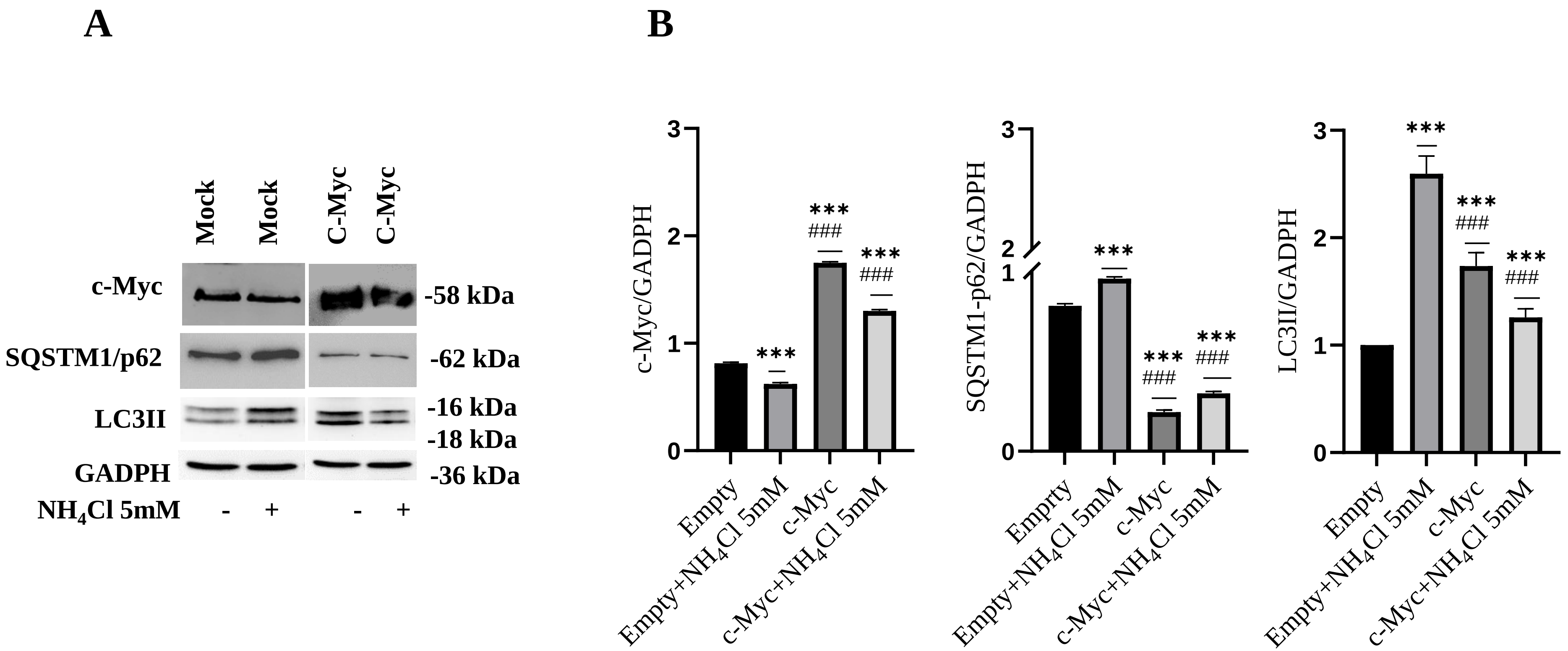

Fig. S4: Overexpression of c-Myc reverse the expression of autophagy markers. **A,B)** Representative immunoblotting and densitometrc analysis of c-Myc, p62/SQSTM1, LC3II in untreated or NH4Cl 5mM treated Huh7 cells overexpressing wild-type c-Myc or a mock conctruct**.** GADPH is used as loading control. (n=2) . The results were presented as means ± standard deviation; values were compared to Empty by one-way ANOVA following Tukey test * $p < 0.0332$ ** $p < 0.0021$ *** $p < 0.0002$ in comparison to empty cells; # $p < 0.0332$ ## $p < 0.0021$ ### $p < 0.0002$ in comparison to empty + NH_4Cl 5mM.
